# Cellular Impact of Micro(nano)plastics on Human Health: A Review

**DOI:** 10.3390/toxics13110913

**Published:** 2025-10-23

**Authors:** Longxiao Liu, Pengcheng Tu, Huixia Niu, Xueqing Li, Xin Gong, Zhijian Chen, Mingluan Xing, Lizhi Wu, Xiaoming Lou

**Affiliations:** 1 School of Public Health, Hangzhou Medical College, Hangzhou 310053, China; 130232024303@hmc.edu.cn; 2 Zhejiang Provincial Center for Disease Control and Prevention, 3399 Binsheng Road, Hangzhou 310051, China; pchtu@cdc.zj.cn (P.T.); niuhuixia1232025@163.com (H.N.); xqli@cdc.zj.cn (X.L.); 202412132405@nuist.edu.cn (X.G.); zhjchen@cdc.zj.cn (Z.C.); mlxing@cdc.zj.cn (M.X.); 3 Center for Disease Control and Prevention of Jinyun County, 89 Cuizhu Road, Jinyun County, Lishui 321400, China; 4 School of Environmental Science and Engineering, Nanjing University of Information Science and Technology, Nanjing 210044, China

**Keywords:** emerging pollutant, microplastics, human health, toxicity

## Abstract

Micro(nano)plastics (MNPs), as a globally emerging environmental pollutant, are now ubiquitous in natural environments and can continuously enter the human body through ingestion, inhalation, and dermal contact. This widespread exposure has raised significant concerns regarding the potential health risks posed by MNPs. Although epidemiological studies are still in the early stages, accumulating in vitro cellular experiments have provided key evidence suggesting that nano- to micro-sized plastic particles can cross physiological barriers in the human body. These particles enter cells via endocytosis or direct penetration through the cell membrane, triggering toxic effects such as oxidative stress, immune responses, mitochondrial dysfunction, and DNA damage, which can potentially lead to cell apoptosis. These findings highlight that the direct interaction between MNPs and human cells could be a core mechanism underlying their potential health hazards. This review systematically summarizes the toxic effects of MNPs exposure on various human cell types, exploring the underlying molecular mechanisms and providing insights for future research into the toxicological impacts of MNPs and their implications for human health risk assessment.

## 1. Introduction

Since the 1950s, global plastic production has increased significantly. From 150 tons in 1950 to 370 million tons in 2019, it is projected that by 2050, global annual plastic production will reach 33 billion tons [1]. The large-scale use of plastics has led to the generation of vast amounts of plastic waste, which accumulates in the environment. Large plastic items exposed to environmental factors such as physical, chemical, and biological processes can degrade into smaller plastic particles, known as MPs [2]. Typically, MPs are defined as plastic particles with a diameter of less than 5 mm [3]. Depending on their origin and method of production, MPs can be categorized into primary and secondary MPs [4]. Primary MPs refer to plastic particles that are intentionally manufactured for use in products, such as the microbeads found in personal care products (PCPs), while secondary MPs are generated through physical, chemical, or biological processes that break down larger plastic items, including plastic fragments, tire wear, and synthetic textile fibers [5]. Additionally, nanoplastics (NPs) are defined as plastic particles with a diameter of less than 1000 nm [6]. These particles come from the further decomposition of MPs, as well as leaks during the production, transportation, and processing of industrial raw materials [7].

**Figure 1:**
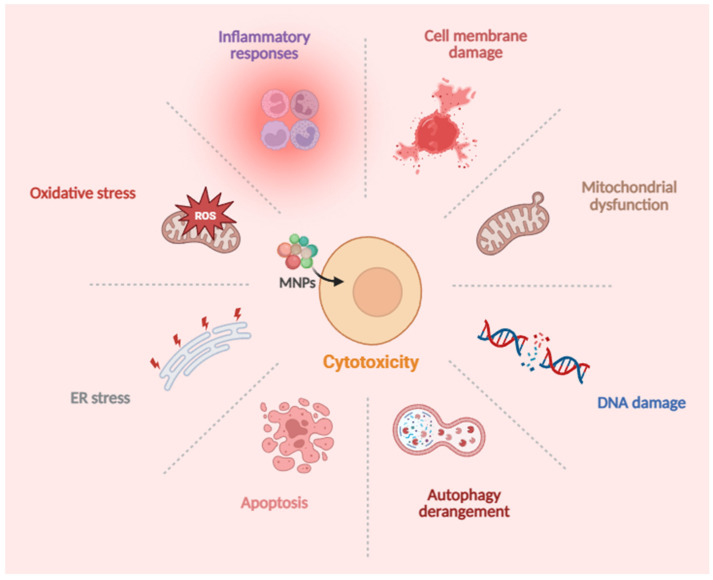
MNPs enter the human body through inhalation, ingestion, and dermal contact, accumulating in tissues and organs. These particles enter cells via endocytosis or direct penetration through the cell membrane, triggering a series of complex cytotoxic effects, including oxidative stress, inflammatory responses, cell membrane damage, mitochondrial dysfunction, DNA damage, autophagy derangement, apoptosis, and endoplasmic reticulum stress. These cytotoxic effects pose potential health risks to humans.

As a globally emerging environmental pollutant, MNPs are now ubiquitous in natural environments and can continuously enter the human body through ingestion, inhalation, and dermal contact [8]. This has raised concerns about the potential health risks posed by MNPs. Although epidemiological studies are still in their early stages, in vitro cellular experiments have accumulated key evidence suggesting that nano- to micro-sized plastic particles can cross physiological barriers in the human body, entering cells via endocytosis or direct penetration through the cell membrane. This interaction triggers toxic effects such as oxidative stress, immune responses, mitochondrial dysfunction, and DNA damage, potentially leading to autophagy derangement and apoptosis [9,10] (Figure 1). These findings indicate that the direct interaction between MNPs and human cells may be a core mechanism underlying their potential health hazards.

In light of this, the present review systematically summarizes the toxic effects of MNPs exposure on different human cell types and their underlying molecular mechanisms, with the aim of providing insights for further research into the potential toxicological mechanisms of MNPs and human health risk assessment.

## 2. Toxic Effects and Molecular Mechanisms of MNPs on Human Cells

Given the challenges of reliably extrapolating data from animal model experiments to humans, this review is based on in vitro cell experiments to systematically summarize the cytotoxic effects and molecular mechanisms of MNPs on various human cell types across physiological systems. The aim is to elucidate potential links between observed pathological phenomena in epidemiological studies and the underlying mechanisms of MNP toxicity.

### 2.1. Respiratory System

The lungs, as the core organ for gas exchange between the body and the external environment, are primary targets for the invasion of atmospheric pollutants. Currently, many studies use human lung epithelial cell lines, such as A549 (type II alveolar epithelial cells) and BEAS-2B (bronchial epithelial cells), as in vitro models for the respiratory system.

Research on the effects of MNPs on the human A549 alveolar type II epithelial cell line indicates that the particle size and surface chemical properties of MNPs are key factors influencing their toxicity. Smaller-sized polystyrene nanoparticles (PS-NPs) are more readily internalized by A549 cells and persist within them, resulting in significant cytotoxic effects [11]. Surface-functionalized PS-NPs also exhibit enhanced cellular internalization and a stronger ability to inhibit cell viability, inducing a reduction in micronucleus (MN) formation [12]. PS-NPs with smaller sizes and positively charged surfaces display stronger toxicity and induce epithelial–mesenchymal transition (EMT) in cells [13]. Moreover, PS-NPs undergo increased toxicity after ultraviolet-induced photodegradation, showing more potent cytotoxic effects than the original nanoparticles, with toxicity correlating positively with exposure duration [14].

MNPs can also induce inflammatory responses in A549 cells, affecting cell proliferation and even leading to apoptosis. Studies have shown that exposure to PS-NPs significantly upregulates the transcription levels of pro-inflammatory cytokines such as IL-6, IL-8, NF-kB, and TNF-α, and promotes the expression of apoptosis-related proteins, including DR5, caspase-3, caspase-8, caspase-9, and Cytochrome C (Cyt c) [11]. The inflammatory responses and apoptosis may further increase the risk of pulmonary fibrosis. One study found that PS-NPs activate NADPH oxidase 4 (NOX4), leading to mitochondrial dysfunction and endoplasmic reticulum (ER) stress, ultimately inducing EMT in A549 cells, a critical step in pulmonary fibrosis. This provides mechanistic evidence for the risk of pulmonary fibrosis due to atmospheric exposure to nanoscale plastics [13]. MNPs also induce oxidative stress and mitochondrial dysfunction. Research has demonstrated that polyethylene terephthalate nanoparticles (PET-NPs) trigger oxidative stress and a decrease in Mitochondrial Membrane Potential (MMP) in A549 cells, with Reactive Oxygen Species (ROS) levels rising continuously and MMP decreasing as exposure dose increases, ultimately reducing cell viability [15].

In the BEAS-2B bronchial epithelial cell line, exposure to PS-MPs induces ROS production, leading to oxidative damage, the release of inflammatory factors (IL-6, IL-8), disruption of lung epithelial barriers dependent on tight junction protein ZO-1, and a reduction in the expression of α1-antitrypsin (AAT) [16]. Additionally, exposure to PS-NPs can trigger apoptosis pathways, causing cell death and impairing alveolar barrier function [17]. These findings suggest that MNPs exposure may increase the risk of lung tissue damage and disease development. Notably, researchers have evaluated the toxicity differences in three types of PS-MPs with different charges on BEAS-2B cells, finding that only the positively charged NH_2_-PS-MPs showed dose-dependent cytotoxicity and significantly increased ROS levels. Further investigation revealed that NH_2_-PS-MPs primarily trigger ER stress through the PERK-EIF2α and ATF4-CHOP pathways, ultimately inducing autophagic cell death in BEAS-2B cells, which causes damage to the bronchial epithelium [18].

Human primary nasal epithelial cells (HNEpCs) are also commonly used as an in vitro model to study the first line of defense in the respiratory system. Studies show that both PS-NPs and PET-NPs are significantly internalized by HNEpC cells, leading to increased ROS production and a reduction in MMP. The exposure also activates the autophagy pathway, as indicated by upregulation of LC3-II and p62 protein expression. Interestingly, despite observing multiple toxic effects, exposure did not result in a significant decrease in cell viability [19,20] (Table 1).

### 2.2. Digestive System

Intestinal epithelial cells serve as the first line of defense in the intestinal mucosa and play a crucial role in maintaining intestinal barrier function. The death of these cells increases intestinal permeability and impairs barrier function, making the intestines more susceptible to various diseases [21]. Wu et al. uncovered the molecular toxic mechanisms of PS-MPs using an in vitro Caco-2 cell model [22]. The study found that PS-MPs inhibited the proliferation of Caco-2 cells in a time- and concentration-dependent manner, disrupting intestinal barrier function. This mechanism was linked to the downregulation of proliferation-related gene expression. Further research revealed that PS-MPs induce oxidative damage and activate NF-κB and MAPK signaling pathways, leading to the release of pro-inflammatory cytokines (IL-1β, IL-8), which produce toxic effects on intestinal epithelial cells [22]. After 48 h of PS-NPs exposure, the expression levels of tight junction proteins Occludin and ZO-1 between Caco-2 cells were significantly reduced, leading to the loss of intestinal barrier integrity. Meanwhile, PS-NPs induced autophagy-dependent cell death by upregulating the pro-apoptotic protein Bax, downregulating the anti-apoptotic protein Bcl-2, and reducing autophagy markers LC3B-II and p62 protein levels [23].

The toxicity of MNPs varies between differentiated and undifferentiated Caco-2 cells. Research shows that short-term exposure (24 or 48 h) to PS-NPs (1–100 μg/mL) did not significantly increase oxidative stress or genotoxicity in undifferentiated Caco-2 cells [24]. Similarly, differentiated Caco-2 cells did not exhibit significant toxic responses at any concentration [25]. Long-term exposure, however, revealed different risk characteristics: undifferentiated Caco-2 cells did not show significant increases in genotoxicity or oxidative stress even after 8 weeks of PS-NPs exposure [26]. However, differentiated Caco-2 cells exposed for 12 days exhibited significant increases in ROS levels, MMP, and mitochondrial respiratory function [27]. These findings suggest that PS-MNPs pose a low acute toxic risk to human gastrointestinal health but may impair redox balance and mitochondrial function with long-term exposure, ultimately compromising intestinal barrier integrity.

The toxicity of MNPs is closely related to their particle size. Smaller particles generally exhibit higher bioactivity and cytotoxicity due to more efficient cellular internalization. In contrast, larger particles, though less readily internalized, are more likely to cause mitochondrial membrane damage. For instance, small particles (50 nm, 100 nm) were more easily internalized by HepG2 cells than larger particles (1000 nm) [28]. In colon epithelial cell models, such as CCD841CoN and HIEC-6 cells, 5 μm PS-MPs were found to damage the mitochondrial membrane, leading to depolarization, with the degree of membrane damage significantly greater than that caused by smaller PS-NPs [29]. A similar study confirmed that 5 μm PS-MPs caused greater disruption to MMP than 0.1 μm PS-NPs [30]. Surface modification is another key factor in regulating MNP toxicity. Amine-modified MNPs, due to their positively charged surface, are more toxic than carboxylated or unmodified particles, potentially inducing apoptosis through membrane disruption, mitochondrial dysfunction, and oxidative stress [28]. Though the mucous layer acts as a physical barrier, its presence does not significantly alleviate the toxicity [31]. Similarly, chlorinated PS-MPs exhibited stronger cytotoxicity in GES-1 cells, significantly inhibiting cell proliferation, altering cell morphology, disrupting membrane integrity, and inducing inflammation and apoptosis [32]. Furthermore, environmental transformation processes may exacerbate the toxicity of MNPs. UV irradiation increases the roughness of PS-MPs surfaces and oxidizes functional groups, thereby compromising the integrity of the Caco-2 cell membrane and gradually decreasing cell viability [33].

Studies have shown that workers in the plastic industry have an increased risk of colon cancer [34]. Recently, a research team studied the effects of MNPs on four different human colorectal cancer cell lines (HT29, HCT116, SW480, and SW620). The study found that all cell lines exhibited significant size- and concentration-dependent uptake of PS-MNPs, with the highest uptake rate in HCT116 cells. During cell division, MNPs were passed to newly formed cells. Moreover, cells exposed to MNPs migrated faster than unexposed cells. When MNPs were present in tumors, they might enhance the migration of cancer cells to other parts of the body, promoting tumor metastasis [35].

In vitro experiments on gastric cells showed that PS-MNPs inhibited cell vitality, increased ROS generation, and activated the P62/Keap1/Nrf2 signaling pathway in a mitochondrial-dependent manner, inducing apoptosis. At the same time, PS-MNPs decreased the expression of tight junction proteins (Occludin, ZO-1, Claudin-4) and adhesion proteins (E-cadherin, β-catenin), disrupting gastric secretion and barrier functions, thus increasing the risk of gastritis or gastric ulcers [36]. Additionally, PS-NPs in GES-1 cells were encapsulated in vesicles, autophagosomes, lysosomes, and lysosomal remnants, destroying cell structure and causing cytotoxicity through oxidative stress, mitochondrial dysfunction, and autophagy, reducing cell proliferation and increasing apoptosis risk [37]. Studies on gastric adenocarcinoma (AGS) cells found that 44 nm NPs accumulated more quickly in the cytoplasm compared to 100 nm NPs, with NPs affecting cell vitality and morphology. The 44 nm NPs strongly induced upregulation of cytokines IL-6 and IL-8 in gastric diseases [38].

MNP-induced hepatocyte apoptosis is a manifestation of liver injury, potentially leading to inflammation, fibrosis, and even cirrhosis [39]. In LO2 cells, PS-MPs induced ER stress via the PERK pathway, leading to oxidative stress, mitochondrial autophagy, and cell apoptosis [40]. PS-MPs also caused cell toxicity in LO2 cells through Ca^2+^ overload and excessive ROS production, resulting in apoptosis [41]. Another study found that MNPs mediated cell apoptosis by increasing oxidative stress in SMMC-7721 cells [42]. Additionally, they upregulate pro-inflammatory cytokine expression via the cGAS/STING pathway in HL7702 cells and participate in mediating hepatic fibrosis [43] (Table 2).

### 2.3. Cardiovascular System

Numerous studies have pointed to a link between MNPs exposure and an increased risk of cardiovascular diseases (CVD), with the potential mechanism being toxicity to vascular cells, including hemolysis, thrombosis, blood coagulation, and endothelial damage. MNPs-induced hemolysis depends on environmental factors, surface modification, and particle size. In a protein-free environment, PS-NPs treatment of human red blood cells (RBCs) showed hemolysis in a dose- and size-dependent manner. Notably, the addition of albumin or whole plasma completely inhibited hemolytic activity when the albumin concentration in the medium reached 0.05% wt [44]. Surface-modified PS-NPs exhibited significant differences in hemolytic activity. Amine-modified PS-NPs (50 nm) caused severe hemolysis, whereas unmodified or carboxylated PS-NPs did not induce significant hemolysis, with hemolytic activity reduced in the presence of 5% plasma [45]. Another study found that PS particles (<5 μm) exhibited hemolytic effects due to their larger surface area, while larger PS particles had no hemolytic effects on RBCs. The study further confirmed that hemolysis was size-dependent, not concentration-dependent [46]. The surface charge of MNPs may also be linked to platelet aggregation and thrombosis. Research indicated that amine-modified PS-NPs could induce procoagulant activity in red blood cells and disrupt platelet membranes through interaction with anionic phospholipids, leading to thrombosis [47,48]. Both carboxylated PS-NPs and amine-modified PS-NPs caused platelet aggregation, whereas unmodified PS-NPs had no effect on aggregation. Amine-modified PS-NPs had a stronger impact on platelet aggregation than carboxylated PS-NPs [45].

Damage to endothelial cell structure and function is associated with atherosclerosis and intracranial aneurysms. When MNPs enter the bloodstream, endothelial cells (EC) are a type of critical target cells that come into direct contact with them. In vitro experiments on human umbilical vein endothelial cells (HUVECs) exposed to different sizes of PS-MNPs showed that PS-NPs (100 and 500 nm) interacted with the cell membrane. Larger particles only bound to the surface, while smaller particles caused membrane damage, increased lactate dehydrogenase (LDH) release, and were absorbed into the cytoplasm, inducing autophagy and autophagosome formation. This may lead to excessive autophagy and necrosis, affecting vascular formation ability [49]. 1 μm PS-MNPs showed low levels of acute toxicity on HUVECs in vitro [50], while 0.5 μm PS-MNPs significantly reduced HUVECs’ activity, angiogenesis, and migration capacity, while inducing autophagy and cell death [51]. These studies suggest that MNPs can induce endothelial cell dysfunction and promote atherosclerotic plaque formation, thereby increasing cardiovascular risks.

The vascular endothelial cadherin (VE-cad), an essential adhesion molecule in endothelial cells, is crucial for maintaining normal endothelial cell function. A study examining NPs’ impact on the vascular endothelial barrier found that barrier disruption was linked not to ROS generation, autophagy, or apoptosis induced by nano-plastics but rather to damage to the VE-cad connections between endothelial cells, disrupting the vascular system barrier [52]. One study evaluated the impact of PS-MPs exposure on premature vascular aging and revealed the molecular mechanisms by which PS-MPs promote premature cardiovascular aging. PS-MPs can be internalized in a dose-dependent manner by HUVECs, significantly increasing intracellular Ca^2+^ levels. Calcium overload induces ROS accumulation in mitochondria, activating CDK5, promoting Lamin A ubiquitin-proteasome degradation, and causing nuclear membrane instability, ultimately leading to endothelial cell aging and damage [53] (Table 3).

### 2.4. Reproductive System

Recent studies have found that MNPs have infiltrated the human male reproductive system, accumulating in the testes and causing a decline in sperm quality. One study explored the effects of different sizes and concentrations of PS-NPs on sperm. The 50 nm particles, at higher concentrations, showed significant toxicity to human sperm, possibly through endocytosis, directly damaging mitochondria and DNA. In contrast, the 100 nm particles had a smaller impact, primarily inducing oxidative stress through interactions with the cell membrane surface. Moreover, the expression of HSP70s was significantly negatively correlated with DNA damage, oxidative stress, and mitochondrial dysfunction, indicating a protective role [54].

Recent research has also explored the toxic effects of MNPs on female reproductive cells. In human ovarian granulosa cells (KGN), PS-NPs reduced cell viability, inhibited cell proliferation, and induced oxidative stress through the activation of the Hippo signaling pathway, leading to changes in cell morphology and even cell apoptosis [55]. In COV434 cells, PS-NPs altered cell morphology, induced oxidative stress, including a decrease in cell viability and MMP, and caused cell cycle arrest and apoptosis [56]. Thus, MNPs may suppress follicle development by inhibiting granulosa cell viability, proliferation, and inducing granulosa cell death, which in turn affects hormone secretion and fertility.

The placental toxicity of MNPs is related to their particle size and chemical composition. A study on human placental cells showed that PS-NPs exhibit size- and surface charge-specific toxicity patterns. Small positively charged PS-NPs were found to be more toxic than their COOH-labeled (negatively charged) or unmodified counterparts. Positively charged NH_2_-labeled PS-NPs interacted with the negatively charged cell membrane, resulting in greater cell toxicity, inhibition of protein kinase A (PKA) activity, oxidative stress, and cell cycle arrest. Further investigation revealed that PS-NPs might increase ROS levels, leading to DNA damage, inflammation, and cell apoptosis, which could potentially affect pregnancy outcomes [57]. While weathering can alter the chemical composition of MNPs, it does not significantly enhance their toxicity to placental cells. A study demonstrated that pristine particles contained a substantially greater number of chemical signals (4411) compared to weathered particles (2150), indicating changes in both the types and quantities of soluble substances during weathering. However, under conditions of acute exposure (100 μg/mL, 24 h), neither pristine nor weathered particles induced significant cytotoxicity [58]. These findings highlight the importance of considering environmental factors in affecting MNP toxicity, as most in vitro studies use original MNPs, overlooking the chemical modifications MNPs undergo in real-world conditions (Table 4).

### 2.5. Urinary System

Numerous studies have investigated the impact of MNPs on kidney cells. In vitro tests with HK-2 cells showed that PS-MPs could enter cells and decrease cell viability, induce mitochondrial dysfunction, ER stress, inflammation, and autophagy [59]. Increased ER stress and cellular inflammation might result from elevated mitochondrial ROS levels and Bad protein levels [60]. PS-MPs exposure suppressed proliferation in human embryonic kidney (HEK 293) cells and human liver (HepG2) cells, altered cell morphology, and reduced the production of glycolytic enzymes, the antioxidant enzyme superoxide dismutase 2 (SOD2) and catalase (CAT). These effects collectively led to excessive ROS production and oxidative stress [61]. In human renal tubular cells and fibroblasts, ROS generation might be associated with extracellular vesicle (EV) production, contributing to ER stress and fibrosis [62]. In HEK 293 cells, PS-MPs exposure induced oxidative stress via the inhibition of heme oxygenase-1 (HO-1), leading to cell toxicity, apoptosis, autophagy, mitochondrial depolarization, and autophagosome formation. Additionally, exposure reduced the expression of proteins maintaining kidney barrier integrity, increasing the risk of acute kidney injury [63] (Table 5).

### 2.6. Nervous System

MNPs exhibit neurotoxicity, reducing cell viability, promoting apoptosis and autophagy, damaging neurodevelopment, altering biomarker expressions related to neurological diseases, and increasing the risk of brain disorders [64]. MNPs entering the human body may travel from the gastrointestinal tract and lungs into the bloodstream, potentially reaching the brain. Studies have detected four types of specific MNPs in human cerebrospinal fluid, indicating that MNPs may enter the central nervous system by disrupting the blood–brain barrier, interfering with neural function, and potentially causing cognitive impairment [65].

Several studies highlight the significant toxic effects of MNPs on human neural cells. Exposure of human neural stem cell line (hNS1) to PS-NPs led to oxidative stress, DNA damage, inflammation, and apoptosis [66]. Research on immortalized human neural stem cells (NSCs) showed that PS-NPs might enter cells through endocytosis and accumulate inside, leading to cell death and reduced cell proliferation through apoptosis [67]. In SH-SY5Y human neuroblastoma cells exposed to PS-NPs, the particles directly damaged neuronal function by disrupting cell membrane integrity, causing a decline in cell viability, retraction of neurites, changes in nuclear morphology, swelling, and leakage of intracellular contents, ultimately leading to necrosis rather than apoptosis [68]. Moreover, exposure also induced mitochondrial dysfunction, activated the AMPK/ULK1 pathway, and led to excessive mitochondrial autophagy, resulting in the death of dopaminergic neurons [69].

Amyotrophic lateral sclerosis (ALS), a neurodegenerative disease, may be associated with the onset of nanoscale plastics. One study found that PS-NPs induced oxidative stress and dysfunction of Hsp70, leading to the abnormal phase transition of TAR DNA-binding protein 43 kDa (TDP-43) and the formation of pathological aggregates in human-derived neurons, causing mitochondrial dysfunction and cell toxicity, potentially related to the mechanism of ALS [70,71].

Microglia, the immune cells of the central nervous system, engulf PS-MPs and trigger inflammatory responses via activation of NF-κB and STAT3 signaling pathways, leading to cell apoptosis. Additionally, PS-MPs induce morphological changes, cell cycle alterations, and gene expression changes in microglia [72].

MNPs may also disrupt cell-to-cell signaling, affecting neural cell function. Aggregation of human α-synuclein is considered a key stage in the pathogenesis of Parkinson’s disease and other neurodegenerative conditions [73]. NPs could increase the aggregation of α-synuclein in synapses, raising the likelihood of Parkinson’s disease [74]. In human cell models of Parkinson’s disease, NPs increased the aggregation of α-synuclein in synapses, induced dopaminergic neuron loss, and exacerbated motor disorders, worsening Parkinson’s symptoms [75] (Table 6).

### 2.7. Immune System

MNPs in the environment can reach the human immune system, entering macrophages and lymphocytes, triggering inflammatory responses and potentially leading to immunotoxicity. Macrophages are the primary target cells for MNPs. The toxicity of MPs to macrophages mainly depends on their ROS generation potential, which is highly positively correlated with the inflammatory level. Additionally, weathered MPs readily adsorb proteins to form a protein corona, which enhances their ability to scavenge ROS and thus reduces their cytotoxicity compared to fresh MPs [76]. When PS-NPs enter macrophages, they induce oxidative stress, reduce cell proliferation and viability, lower MMP, and cause DNA damage, leading to cell apoptosis [77]. PS-NH_2_ can directly activate the NLRP3 inflammasome in THP-1 cells to release IL-1β, inducing inflammation and cytotoxicity. PET, polyacrylonitrile (PAN), and nylon (PA6) induce IL-8 secretion in NLRP3^−/−^ cells, triggering inflammation [78]. PS-NPs may also influence lipid droplet (LD) accumulation inside macrophages through oxidative stress, altering lipid metabolism and potentially leading to macrophage differentiation into foam cells [79]. This could promote the formation of atherosclerotic plaques. However, some studies suggest that PS-MPs do not interfere with the differentiation or activation of human macrophages [80].

MNPs may also have potential genetic and cytotoxic effects on human peripheral blood lymphocytes. Exposure of peripheral blood lymphocytes to PS-MNPs showed a dose-dependent decrease in the mitotic index (MI) and nuclear division index (NDI), along with increased frequencies of MN, nucleoplasmic bridges (NBP), and nuclear buds (NBUD) [81,82]. A study on PVC-MNPs exposure found significant differences in lymphocyte responses between humans and fish (Yellowfin tuna). Human blood lymphocytes showed dose-dependent cytotoxicity after just 3 h of exposure, with elevated ROS levels, increased lysosomal membrane permeability, MMP collapse, and metabolic imbalances. In contrast, fish lymphocytes showed no significant changes [83]. This study suggests that human lymphocytes are more sensitive to PVC, possibly due to differences in metabolic enzymes or antioxidant capacities between species.

Furthermore, MNPs can activate immune cells (PBMCs), disrupt immune homeostasis, and stimulate the release of inflammatory factors (IL-6 and TNF-α). A study demonstrated that exposure of PBMCs to MPs resulted in immune activation, with ABS and PVC specifically inducing the production of IL-6 and TNF-α, respectively [84]. High concentrations (1000 μg/mL) of PS microfragments amplified acute inflammatory responses by 20 times, significantly increasing the release of IL-6 and TNF-α in PBMCs, and induced cell death over long-term exposure (4 days) [85]. The toxicity of PP was related to particle size and concentration, with smaller particles at higher concentrations more likely to induce inflammation and immune responses in PBMCs, releasing IL-6 and TNF-α, along with increased histamine secretion, suggesting a potential allergic reaction [86]. MNP shape also influences toxicity. HDPE microbeads induced immune responses at high concentrations, while LDPE, with its irregular shape, induced more significant inflammation, releasing IL-6 and TNF-α in a concentration-dependent manner [87]. Irregularly shaped PVC particles enhanced the release of pro-inflammatory cytokines (IL-6, TNF-α) in primary human monocytes and dendritic cells [88] (Table 7).

## 3. Limitations of Human Cell Models

In vitro human cell models have become effective tools for revealing cellular-level mechanisms of life and disease due to their advantages, such as simulating specific tissue microenvironments, elucidating molecular mechanisms, and complying with ethical standards. However, primary cells or immortalized cells isolated from the human body may exhibit significant differences in physiological responses between in vivo and in vitro environments.

Primary cells and immortalized cell lines, derived from human tissues, maintain high physiological relevance. These models are relatively simple to operate, easy to standardize, and cost-effective to maintain. Researchers can quickly master their culture techniques and exert high control over the cellular environment, making them suitable for high-throughput applications. However, the in vitro culture environment differs significantly from the true microenvironment in the human body. After isolation, cells lose the regulation from neurohumoral factors and the intercellular interactions present in vivo. They also lack the dynamically stable environment and natural biomechanical forces, such as fluid flow or periodic contraction, that influence cellular behavior and disease processes. This results in a reduced translational value for in vitro cell cultures.

Additionally, in vitro cells are inherently unstable. During culture, they may undergo artificial mutations, losing some biological characteristics, leading to abnormal cellular behavior that cannot be accurately predicted in vivo cell function. Moreover, cell cultures are often limited to cancerous or immortalized cell lines, which differ fundamentally in their proliferation characteristics and metabolic pathways compared to healthy human cells, further reducing their predictive accuracy.

To enhance the predictive accuracy of cell models, researchers have developed advanced in vitro three-dimensional (3D) culture technologies, such as spheroid models [89] and organ-on-a-chip models [90]. These models aim to recreate tissue microenvironments more closely resembling in vivo conditions, allowing for more accurate simulations of the impact of MNPs on human organs. Since organoid models are constructed using human-derived cells, the information they carry is consistent with human biology, overcoming the species differences encountered in animal models. Furthermore, organoid technology raises fewer ethical concerns and can simulate the chronic toxicity of MNPs to explore potential health risks to humans [91].

Currently, numerous organoid models have been applied to assess the health risks of MNPs to human organs, including the gut [92,93], liver [94,95], brain [96,97] and kidney [98,99]. However, existing organoid models face limitations in areas such as culture conditions, functionality, longevity, heterogeneity, and reproducibility. Additionally, they lack components like blood vessels, nerves, and immune cells, making it challenging to analyze the complex biological effects of MNPs [100].

## 4. Conclusions and Future Perspectives

In vitro cell experiments show that the toxicity of plastic particles depends on the size, shape, type, surface charge, and environmental weathering of MNPs. First and foremost, particle size and shape are the primary determinants. Specifically, smaller particles induce stronger toxic effects, owing to the fact that they have a greater ability to penetrate cellular membranes and biological barriers. Similarly, fibrous and irregular fragments exhibit greater cytotoxicity than spherical particles, as their sharp tips or edges can cause sustained mechanical stimulation to cell membranes, thereby triggering more pronounced inflammatory responses and cellular damage. In addition, different types of MNPs have varying degrees of toxicity toward the same cell. Furthermore, surface charge and environmental weathering significantly modulate toxicological outcomes. For instance, for MNPs of identical size, shape, and polymer type, positively charged particles typically exhibit higher toxicity than negatively charged ones, which is attributable to enhanced electrostatic interactions with negatively charged cell membranes. While environmental weathering can augment MNP toxicity by altering surface chemistry, under physiologically relevant conditions, these changes may increase the protein adsorption capacity of the particle surface, leading to the formation of a “protein corona” that can mitigate their toxic effects. Additionally, dose and exposure duration remain critical factors, with toxicity demonstrating clear dose- and time-dependent relationships.

However, current research predominantly relies on laboratory-synthesized standard particles, which are typically uniform in size and typically spherical, composed of single polymers, and are often assessed using relatively high exposure doses in experimental designs. In contrast, environmental MNPs exhibit high complexity, generally demonstrating a continuous size distribution from micron to nano scales, diverse morphologies including fibers, fragments, and irregular particles, and frequently existing as mixtures of multiple polymers. Consequently, existing laboratory models may lead to deviations in ecological risk assessment outcomes when simulating real-world MNPs exposure.

To enable more accurate prediction of the health impacts of MNPs, future studies should prioritize the use of MNP samples that more closely reflect environmentally relevant features, along with exposure regimens based on actual environmental detection concentrations, thereby allowing experimental designs to better mimic real-world conditions.

## Figures and Tables

**Table 1:** The toxic effects of MNPs on human respiratory system cells.

Cell Model	MNP Type	Size	Dose	Time	Toxic Effect	Ref.
A549	PS	25, 75 nm	2.5–300 μg/mL	12 h	Cell viability ↓ Inflammatory responses ↑ Cell apoptosis ↑ Cell cycle S-phase arrest Disturb gene transcription and protein expression	[11]
	PS, NH_2_-PS, COOH-PS	2 μm, 80 nm	0–400 μg/mL	24 h	Cell viability ↓ Genotoxicity ↑ Oxidative stress ↑	[12]
	PS	20, 50 nm	10–160 μg/mL	24 h	Cell viability ↓ ROS generation ↑ MMP ↓ ER stress ↑ Mitochondrial dysfunction	[13]
	PS, UV PS	100 nm	5–100 μg/mL	24 h	Cell viability ↓ Oxidative stress ↑ Membrane damage ↑ Mitochondrial dysfunction Genotoxic and oxidative DNA damage	[14]
	PET	164, 190 nm	0.10–300 μg/mL	24 h	Cell viability (0.10–0.98 μg/mL ↑ 4.92–196.79 μg/mL ↓) Oxidative stress ↑ MMP ↓ Cell apoptosis ↑	[15]
BEAS-2B	PS	1.72 ± 0.26 μm	1–1000 μg/cm^2^	24, 48 h	Cytotoxicity ↑ Inflammatory responses ↑ ROS accumulation ↑ Barrier dysfunction	[16]
	PS, NH_2_-PS, COOH-PS	100 nm	25–400 μg/mL	24 h	Cell viability (NH_2_-PS-MPs) ↓ ROS generation ↑ ER stress ↑ Inflammatory responses ↑ Autophagic cell death ↑	[18]
HPAEpiC, BEAS-2B	PS	40 nm	24, 48, 96 μg/mL	24 h	Cell viability ↓ Oxidative Stress ↑ Inflammatory adverse response ↑ Cell apoptosis ↑ Alter the gene expression Alveolar Epithelial Barrier damage Pulmonary dysfunction	[17]
HNEpCs	PS	50, 500 nm	0.5–100 μg/mL	24 h	Oxidative Stress ↑ Loss of MMP Disturbance to the autophagy pathway	[19]
	PET	62.38 nm ± 3.51	0–100 μg/mL	24 h	iROS generation ↑ Loss of MMP Alter the autophagy pathway	[20]

Note: ↑—increased, ↓—decreased.

**Table 2:** The toxic effects of MNPs on human digestive system cells.

Cell Model	MNP Type	Size	Dose	Time	Toxic Effect	Ref.
Caco-2	PS	5 μm	10^−1^–10^−5^ mg/mL 12.5, 25, 50 mg/L	24, 48 h	Cell viability ↓ Oxidative Stress ↑ Epithelial cell injury and alterations to intestinal barrier function Change the transcription level of genes	[22]
	PS, PS-COOH, PS-NH_2_	100 nm	30, 60, 120, 240, 480 μg/mL	24, 48, 96 h	Cell proliferation ↓ Cell apoptosis ↑ Autophagic cell death ↑ Disrupt the intestinal barrier function	[23]
	PS	0.1, 5 μm	1, 10, 20, 40, 50, 80, 200 μg/mL	12 h	ROS generation ↑ Mitochondrial depolarization	[30]
	PS	1.0–1.9 μm	0, 50, 500, 1000 μg/mL	24 h	Cell viability ↓ Cell membrane damage ↑	[33]
HepG2	PS(amine, carboxyl and non-functionalize)	50–5000 nm	0.1–100 μg/mL	1–24 h	Cell viability ↓ Inflammatory response ↑ Cell apoptosis↑	[28]
CCD841CoN HIEC-6	PS	0.1–5 μm	0, 12.5, 25, 50, 100 μg/mL	0.5, 1, 4, 8, 12, 24 h	Oxidative Stress ↑ Membrane damage ↑ Mitochondrial depolarization	[29]
GES-1	PS, Cl_2_-PS	213.7 ± 8.2 nm	1, 10, 20, 50, 100 mg/L	48 h	Cell viability ↓ Mitochondria-dependent apoptosis ↑ Cell membrane damage ↑ Oxidative stress ↑ Inflammatory response ↑ Mitochondrial dysfunction Alter cell morphology	[32]
	PS	50, 250 nm	0, 20, 40, 80 μg/mL	24, 48 h	Cell viability ↓ Oxidative Stress ↑ MMP and ATP level ↓ Mitochondria-dependent apoptosis ↑ Inhibit gastric juice secretion and mucus secretion Disrupt gastric barrier function Mitochondria dysfunction	[36]
	PS	60 nm	50 μg/mL	2, 4, 6, 12, 24, 48 h	Cell proliferation ↓ Apoptosis ↑ Autophagy ↑	[37]
LO2	PS	5 μm	0.5 mg/mL	24 h	ER stress ↑ Oxidative Stress ↑ Apoptosis ↑ MMP ↓ Mitochondrial fission, apoptosis, and mitophagy ↑	[40]
	PS	5, 10 μm	0.2, 0.4, 0.6 mg/mL	24 h	ROS ↑ Apoptosis↑ S phase arrest	[41]
SMMC-7721	PS	500 nm, 50 μm	2, 5, 10, 20, 50, 100 μg/mL	24 h	Cell viability ↓ Cell membrane damage and apoptosis ↑ Cell morphological alteration	[42]
HL7702	PS	0.1, 1 μm	0.1, 1 mg/L	24 h	Nucleus damage and micronucleus formation Mitochondria DNA damage	[43]

Note: ↑—increased, ↓—decreased.

**Table 3:** The toxic effects of MNPs on human cardiovascular system cells.

Cell Model	MNP Type	Size	Dose	Time	Toxic Effect	Ref.
RBC	PS	50, 107, 250 nm	50, 150, 250, 350, 500 μg/mL	1 h	Hemolysis ↑	[44]
	PS	50 nm	0–1000 μg/mL	20 min	Platelet aggregation ↑	[45]
	PS	50, 100, 1000 nm	10–500 μg/mL	3, 24 h	Hemolysis ↑ Cell adhesion and thrombin generation ↑ Morphological and membrane changes	[47]
HUVEC	PS	100, 500 nm	0, 5, 10, 25, 50, 100 μg/mL	0–48 h	Cell viability ↓ Lysosomal damage Autophagosomes accumulation Cell membrane damage	[49]
	PS	0.5, 1, 5 μm	0, 20, 40, 60, 80, 100 μg/mL	24, 48, 72 h	Cell viability ↓ Angiogenic signaling pathway ↓ Wound healing and cell migration ↓ Cell senescence ↑ Autophagy and necrosis ↑ Angiogenic tube formation ↓	[51]
	PS	21.2 ± 3.5 nm	0–0.5 mg/mL	1, 3, 6 h	Endothelial leakiness ↑	[52]
	PS	1 μm	0, 5, 10, 25, 100 μg/mL	48 h	Cell viability (100 μg/mL ↓)	[50]
HCAECs, HUVEC	PS	1 μm	0.1, 0.3, 0.6, 0.9 mg/mL	90 min	Cell senescence ↑ Oxidative Stress ↑ Lamin A ↓	[53]

Note: ↑—increased, ↓—decreased.

**Table 4:** The toxic effects of MNPs on human reproductive system cells.

Cell Model	MNP Type	Size	Dose	Time	Toxic Effect	Ref.
Semen	PS	50, 100 nm	0.1, 0.5, 1 μg/mL	30 min	Motility ↓ Acrosomal damage ↑ Oxidative stress ↑ DNA fragmentation ↑ Mitochondrial activity ↓	[54]
KGN	PS	15–38 nm	50, 100, 200 μg/mL	24, 48, 72 h	Cell proliferation ↓ Oxidative stress ↑ Cell apoptosis ↑	[55]
COV434	PS	50 nm	50, 100, 150, 200 μg/mL	24 h	Cell viability ↓ MMP ↓ Oxidative stress ↑ Cell apoptosis ↑	[56]
JEG-3	PS, PS-NH_2_, PS-COOH	25, 50, 100, 500 nm	0, 20, 39, 78, 156, 313, 625, 1250, 2500, 5000 μg/mL	24 h	Cell viability ↓ PKA activity ↓ Oxidative stress ↑ DNA damage ↑ Inflammation and apoptosis ↑ Cell cycle arrest	[57]
BeWo b30	PS, HDPE	PS: 0.05–10 μm HDPE: 0–80 μm	0.1–100 μg/mL	1–48 h	Membrane damage (100 μg/mL) Alter gene expression	[58]

Note: ↑—increased, ↓—decreased.

**Table 5:** The toxic effects of MNPs on human urinary system cells.

Cell Model	MNP Type	Size	Dose	Time	Toxic Effect	Ref.
HK-2	PS	2 μm	0.025, 0.05, 0.1, 0.2, 0.4, 0.8 mg/mL	0–48 h	Mitochondrial ROS ↑ ER Stress ↑ Inflammation ↑ Autophagy ↑ Apoptosis ↑ Mitochondrial Dysfunction	[59]
HEK 293	PS	1 μm	0.05, 5, 10, 25, 50, 75, 100 μg/mL	0–72 h	Cell Morphological Changes Cellular Proliferation ↓ Metabolic Activity ↓ ROS Levels ↑	[61]
HK-2	PS	Water: 2121.3 nm K-SFM: 2209.0 nm	0.4, 0.8 mg/ml	0–24 h	Extracellular vesicle production ↑ ER stress-related proteins ↑ ROS production ↑ Fibrosis-related proteins ↑	[62]
HEK293	PS	3.54 ± 0.39 μm	3–300 ng/mL	24 h	Inflammatory responses ↑ Apoptosis and autophagy ↑ Barrier integrity ↓ Oxidative and inflammatory ↑	[63]

Note: ↑—increased, ↓—decreased.

**Table 6:** The toxic effects of MNPs on human nervous system cells.

Cell Model	MNP Type	Size	Dose	Time	Toxic Effect	Ref.
hNS1	PS	30 nm	0.5, 2.5, 10 μg/mL	4 days	Oxidative stress ↑ Cellular Stress ↑ DNA Damage ↑ Inflammatory response ↑ Apoptotic ↑	[66]
NSCs	PS	30 nm	0.5, 2.5, 10 μg/mL	1–4 days	Apoptosis ↑ Cell proliferation ↓	[67]
SH-SY5Y	PS	50 nm	27.6, 138, 690 μg/mL	24 h	Neurite outgrowth ↓ Morphology alteration and swelling of the nuclei Spilling of intracellular components	[68]
	PS	50 nm	0.5, 5, 50, 500 μg/mL	48 h	ROS levels ↑ Cell death ↑ Dopaminergic neuron ↓ Mitophagy ↓ Mitochondrial dysfunction Disrupt mitochondrial respiration	[69]
HMC-3	PS	0.2, 2, 10 μm	1, 5, 10 μg/mL	24, 48, 72 h	Microglial activation ↑ Apoptosis ↑ Immune responses ↑ Microglial morphological change	[72]

Note: ↑—increased, ↓—decreased.

**Table 7:** The toxic effects of MNPs on human immune system cells.

Cell Model	MNP Type	Size	Dose	Time	Toxic Effect	Ref.
THP-1	PP, PS	100 μm	625–20,000 particles/mL	24 h	ROS generation ↑ Inflammation ↑	[76]
	PS	100–450 nm	50–500 μg/mL	4, 24, 48, 72, 96 h	Cell viability and proliferation ↓ Oxidative stress ↑ Apoptosis ↑ DNA damage ↑ Morphology changes Mitochondrial membrane damage	[77]
Peripheral lymphocytes	PS	50 nm	500, 1000, 2000 μg/mL	48 h	Cell viability ↓ Hemolysis ↑ Mitotic Index and nuclear division index ↓ Micronuclei frequency and cytostasis ↑	[81]
	PE	10–45 μm	25, 50, 100, 250, 500 μg/mL	48 h	DNA Damage ↑ Micronucleus formantion ↑ Nucleoplasmic bridge formation ↑ Nuclear bud formation ↑	[82]
Human lymphocytes	PVC	0.16–1.82 μm	24, 48, 96 μg/mL	3 h	ROS formation ↑ Lysosomal membrane injury ↑ GSH ↓ Lipid peroxidation ↑ MMP collapse	[83]
PBMCs	PVC, ABS	25–75, 75–200 μm	10, 100, 1000 μg/mL	1–5 days	Immune responses ↑	[84]
	PS	5–25, 25–75, 75–200 μm	5, 25, 75, 200 μg/mL	1–4 days	Immune responses ↑ Inflammation ↑ Cell death ↑ Cell membrane damage ↑ Hemolysis ↑	[85]

Note: ↑—increased, ↓—decreased.

## Data Availability

No new data were created or analyzed in this study. Data sharing is not applicable to this article.
